# Ultrahigh sensitive refractive index nanosensors based on nanoshells, nanocages and nanoframes: effects of plasmon hybridization and restoring force

**DOI:** 10.1038/s41598-021-81578-w

**Published:** 2021-01-22

**Authors:** MirKazem Omrani, Hamidreza Mohammadi, Hamidreza Fallah

**Affiliations:** 1 Department of Physics, University of Isfahan, P.O. Box 81746-7344, Isfahan, Iran; 2 Quantum Optics Research Group, University of Isfahan, Isfahan, Iran

**Keywords:** Sensors and biosensors, Nanophotonics and plasmonics

## Abstract

In this study, the effect of the plasmon hybridization mechanism on the performance and refractive index (RI) sensitivity of nanoshell, nanocage and nanoframe structures is investigated using the finite-difference time-domain simulation. To create nanocage structure, we textured the cubic nanoshell surfaces and examined the impact of its key parameters (such as array of cavities, size of cavities and wall thickness) on the nanocage's RI-sensitivity. Synthesis of the designed nanocages is a challenging process in practice, but here the goal is to understand the physics lied behind it and try to answer the question “Why nanoframes are more sensitive than nanocages?”. Our obtained results show that the RI-sensitivity of nanocage structures increases continuously by decreasing the array of cavities. Transforming the nanocage to the nanoframe structure by reducing the array of cavities to a single cavity significantly increases the RI-sensitivity of the nanostructure. This phenomenon can be related to the simultaneous presence of symmetric and asymmetric plasmon oscillations in the nanocage structure and low restoring force of nanoframe compared to nanocage. As the optimized case shows, the proposed single nanoframe with aspect ratio (wall length/wall thickness) of 12.5 shows RI-sensitivity of 1460 nm/RIU, the sensitivity of which is ~ 5.5 times more than its solid counterpart.

## Introduction

Noble metallic nanoparticles have received vast applications in the fields of sensors^1,2^, photodetectors^3^, plasmonic solar cells^4–6^, cancer treatment and therapy^7,8^, etc. due to one of their capabilities; localized surface plasmon resonances (LSPRs) generation, and ability of light localization in nanoscale^9^. LSPR is the result of collective oscillations of conduction electrons on the surface of metallic nanoparticles which are induced by electromagnetic fields of the incident light^10^. Generation of LSPR enables the strengthening of electromagnetic fields, absorption and scattering of light, which depends on the shape, size, and chemical composition and environment of the nanoparticles^1,11–13^. The dependence of the LSPR properties of metallic nanoparticles on their surrounding medium is indeed the basic principle for the use of nanoparticles for refractive index (RI) nanosensors. A red- or blue-shift phenomenon may occur in LSPR wavelength when the refractive index of the local environment is changed. This feature of metallic nanoparticles allows us to design optical nanosensors for the detection of the chemical changes such as protein interactions^14^, antibodies^15^ in molecular dimensions for applications involved in biomarker for Alzheimer’s disease^16^ and so on.

The RI-sensitivity can potentially be tuned and controlled by key parameters of nanoparticles such as shape and size to achieve high sensitivity nanosensors^11,12^. In this regard, chen et al. investigated the solid gold nanostructures including nano-rods, nano-cubes, nano-spheres, nano-bipyramides and nano-branches and reported their RI-sensitivity in the range of 44–703 nm/RIU; the lower sensitivity is for 50 nm nano-sphere and the upper bound is reached by 80 nm nano-branch^11^. Khan et al. introduced aspect ratio (R) as a key parameter that controls the solid nanoparticle sensitivity (S) following an empirical equation, S = 46.87 × R + 109.37. They believe that the correlation between shape and sensitivity is much weaker than that between aspect ratio and sensitivity^12^. Reviews show that there are two methods to increase the RI-sensitivity of solid nanoparticles: lengthen the nanoparticles and sharpen its apexes^11,12^.

On the other hand, hollow nanostructures showed that they can achieve ultra-high sensitivities thanks to their better plasmonic properties, based on the plasmon hybridization mechanism^17,18^. To describe the plasmon hybridization mechanism, Prodan et al. considered a nanoshell including an inner cavity and an outer spherical surface, having different resonance frequencies^19^. The cavity plasmons interact with the sphere plasmons thanks to finite thickness of the nanoshell. The strength of this interaction could be adjusted by manipulating the nanoshell thickness. Due to this interaction, the plasmonic oscillations of the nanoshell is split into symmetric and asymmetric oscillations, symmetric oscillation occurs at smaller frequencies and hence has lower energy than the asymmetric ones. Unlike asymmetric oscillation, which is considered as a dark mode and does not couple to the far-field radiation, the symmetric oscillations are coupled with the external optical fields and have greater RI-sensitivity than asymmetric plasmonic oscillations^19^.

The hybridization model has a significant role in the RI-sensitivity improvement of metallic nanoparticles and its validity is tested by quantum mechanical calculations and also by FDTD simulations^18,20^. The influence of nanoshell thickness on the plasmonic hybridization mechanism is studied by Halas^19^. Her results show that the energy gap between two modes of hybrid surface plasmons (symmetric and asymmetric modes) increases as the nanoshell thickness decreases and hence frequency shift (with respect to solid nanoparticle) is larger for thinner nanoshells. Accordingly, the spherical nanoshells are the simplest structures in complex hollow structures which hybridization model could describe.

Progressive developments in the nanoparticle synthesis^21,22^ have introduced complex nanostructures with high degree of RI-sensitivity^23,24^. Among them, high potential structures of nanocage and nanoframe can be mentioned. In this way, Yugang Sun and Younan Xia have recorded a sensitivity of 408.8 nm/RIU for the nanocage structure with a 50 nm wall length and a 4.5 nm wall thickness^25^. Also, Mahmoud A. Mahmoud and Mostafa A. El-Sayed synthesized the gold nanoframes with different wall thicknesses and reported RI-sensitivity of 620 nm/RIU for nanoframes with 51 nm wall length and 10 nm wall thickness^24^. They develop an equation for estimating the sensitivity of nanoframes as function of aspect ratio (ratio of wall length to wall thickness) in order to make it possible to compare nanoframes and nanocages synthesized by Sun and his colleague in the same aspect ratio. The results showed a ~ threefold sensitivity of nanoframes compared to nanocages, but the reason for the superiority of nanoframes over nanocages has remained unanswered so far.

In this work, motivated by finding a reason for the more RI-sensitivity of nanoframes than nanocages, we have numerically investigated the LSPR properties and RI-sensing capabilities of the plasmon hybridization based cubic nanoshells, nanocages, and nanoframes by using FDTD simulation. In this way, we have first examined the effect of shell thickness on the plasmonic response of SiO_2_@Au core–shell nanocubes. Then by texturing the surfaces of optimized Au shell with array of cavities and removing the silica core, we have investigated the effect of key parameters (such as array of cavities, size of cavities, and wall thickness) on LSPR properties and RI-sensitivity of created nanocages. We have shown that the nanocages are less sensitive than nanoframes due to the simultaneous presence of symmetric and asymmetric oscillations in nanocage structures at the plasmon resonance mode. Finally, we have presented a linear equation for estimating the RI-sensitivity of nanoframes as a function of aspect ratio in a wide range.

## Results and discussion

The nanoshell structures have better plasmonic properties than their solid counterparts due to the plasmon hybridization mechanism. When a hollow structured nanoparticle illuminated by the electromagnetics wave, the electrons of inner and outer surfaces show different plasmonic oscillation frequencies. These oscillations could interact with each other. The strength of the interaction is controlled by the nanoshell thickness. There exist two regimes of interactions, the symmetric and asymmetric modes. The symmetric mode couples to the optical fields and is sensitive to the variation of the medium’s refractive index. The plasmon hybridization theory predicts that reducing the nanoshell causes the symmetric mode to oscillate at lower frequencies (this is the origin of red-shift phenomenon)^19^.

**Figure 1:**
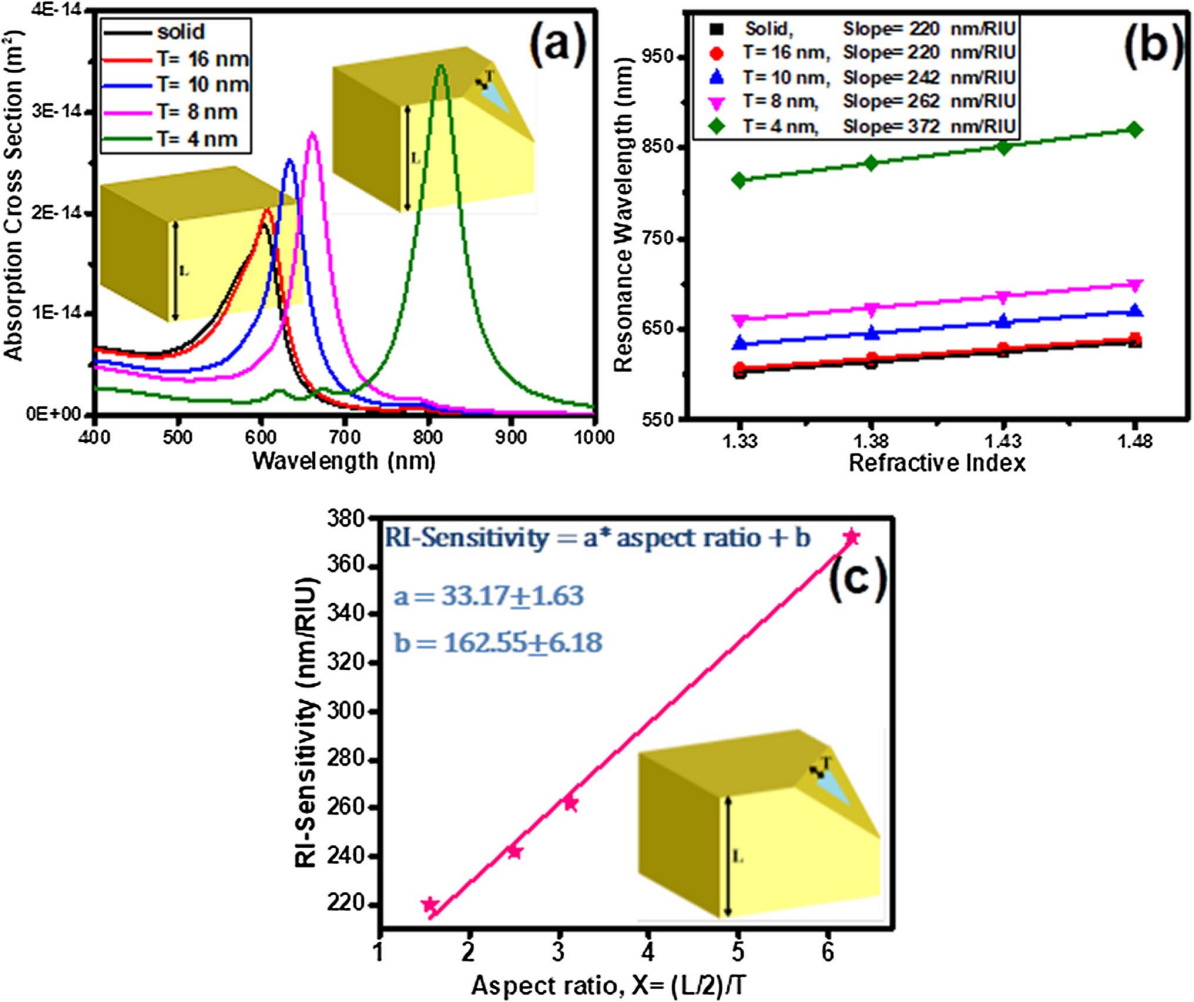
(**a**) Absorption spectra of silica@gold core–shell nanoparticles with various shell thickness, T (n = 1.33). (**b**) The LSPR shift of cubic nanoshells with various shell thickness (T) versus different refractive index of surrounding medium. (**c**) The RI-Sensitivity shift of cubic nanoshells versus different aspect ratio, X (Half the total size (L/2) -to- shell thickness (T)) ratio). A fit to the data with linear equation S = a × X + b. Browser-based version of SketchUp, https://app.sketchup.com, and Origin 2020 version, https://www.originlab.com, have been used for drawing images and making figures, respectively.

The effect of plasmonic hybridization on the sensitivity of 50 nm gold cubic nanoparticles during the conversion of its structure from solid to silica-gold core–shell, has been studied using FDTD simulation (Fig. 1). As the Au shell thickness decreases, the nanoparticle absorption spectrum peak shifts from the visible spectral region to higher wavelengths in the near-infrared spectral region and the intensity also increases (Fig. 1a), stemming from the coupling strengthening of the inner and outer surfaces plasmons. The charge density distribution pattern at the Au nanosolid and nanoshell surfaces in the resonance mode shows the accumulation of charge at the edges and corners of cubic nanoparticles as well as the hybridization oscillations of coupled plasmons at the inner and outer surfaces of the nanoshells (see figure S1 in supplementary materials). Unlike spherical nanoshells, which fully symmetric plasmon oscillations occurs at their coupled surfaces, cubic nanoshells show the simultaneous existence of symmetric and asymmetric oscillations on vertical and parallel surfaces with respect to the polarization of the incident light, respectively. This can act as a limiting factor in nanoparticle RI-sensitivity within the structure. The RI-sensitivity is defined by the line slope of resonance wavelength versus the refractive index of the medium. Figure 1b shows the investigation of the effect of the shell thickness on RI-sensitivity of silica core-gold shell nano-cubes. This figure reports the RI-sensitivities of 220, 242, 262 and 372 nm/RIU for the shell thickness (T) of 16, 10, 8 and 4 nm, respectively. Figure 1c shows the linear increase in the sensitivity of the core–shell silica-gold nanoparticles by increasing the aspect ratio, X (Half the total size (L/2) -to- shell thickness (T)) ratio), which is formulated as follows:

1 $$S = \frac{\delta \lambda }{{\delta }} = \left( {33.17 \pm 1.63} \right)\frac{L/2}{T} + \left( {162.55 \pm 6.18} \right),$$

here, the core–shell nanocube with a shell thickness of 4 nm and aspect ratio of 6.25 represented the highest sensitivity of 372 nm/RIU. Plasmonic coupling not only increases the RI-sensitivity of the cubic core–shell SiO_2_@Au nanoparticles to changes in the refractive index of the environment, but also amplifies the generated near-field, increases its decay length as well as increases the plasmons lifetime (Figure S2).

**Figure 2:**
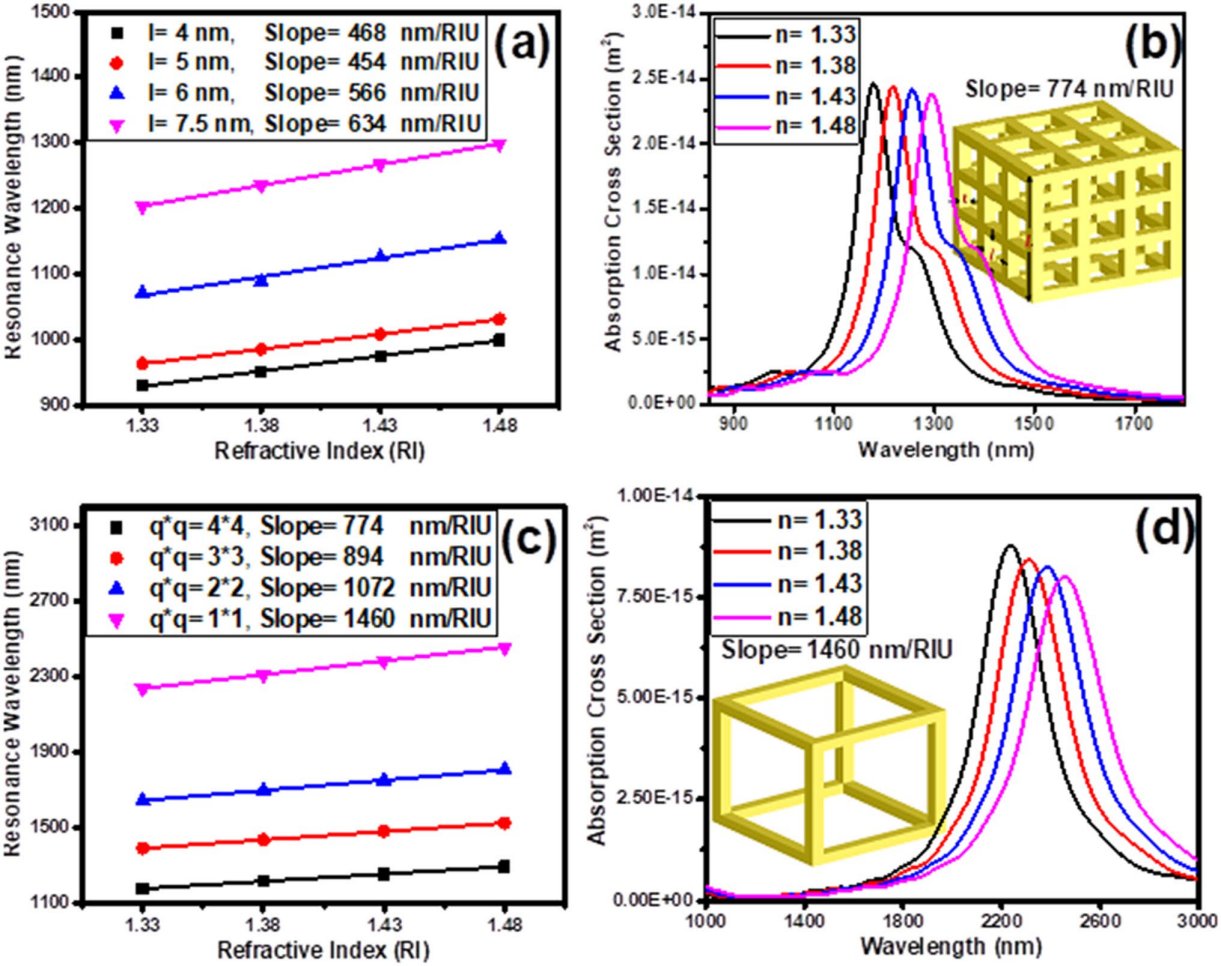
(**a**) Resonance wavelength shift of core-texture shell SiO_2_@Au nanocages versus refractive index of surrounding medium for different etch lengths, *l* (cavities array are 4*4). (**b**) Absorption cross section of etched core-texture shell optimized cubic nanocage (*T* = 4 nm and *l* = 7.5 nm (g = 4 nm)) versus refractive index of surrounding medium. (**c**) Resonance wavelength shift of etched core-texture shell versus different refractive index of surrounding medium for different number of cavities array (**d**) Absorption cross section of optimum cubic nanocage (nanoframe) versus refractive index of surrounding medium. Browser-based version of SketchUp, https://app.sketchup.com, and Origin 2020 version, https://www.originlab.com, have been used for drawing images and making figures, respectively.

In the following, the effect of key parameters (such as the array of cavities created on the surfaces of the gold nanoshell, size of the cavities and wall thickness) on the plasmonic hybridization mechanism and RI-sensitivity of the nanocages are investigated and presented in Fig. 2. The square cavity size ($l$) is optimized by creating 4*4 array of cavities in the core–shell nanocube surfaces, as shown in Fig. 2a. Increasing the size of the cavities (reducing the wall thickness) from 4 to 7.5 nm (from 6.8 to 4 nm), increases the sensitivity of the nanocages from 468 to 634 nm/RIU, which can be related to the strengthening of plasmons interaction in the walls. Further improvement of RI-sensitivity can be achieved by enhancing the contact surface of nanoparticle with its surrounding medium. This is possible by etching the SiO_2_ core. Figure 2b reveals this fact and shows the red-shift of the absorption spectrum peak of empty core nanocage (with the wall thickness of 4 nm and the 4*4 cavities array) as a function of the environment refractive index increment. This figure shows that the RI-sensitivity of nanocage is increased from 634 (for silica filled core 4*4 nanocage) to 774 nm/RIU (for empty core 4*4 nanocage). Also, the sensitivity of nanocages with 4*4, 3*3, 2*2, and 1*1 cavity arrays are investigated in Fig. 2c (wall thickness has been fixed.). The results show that by reducing the array of cavities from 4*4 to 1*1, the sensitivity of nanocages increases significantly from 774 to 1460 nm/RIU. The nanocage with a 1*1 cavity array, i.e. the nanoframe, has recorded the highest sensitivity. The response of its absorption spectrum as a function of the refractive index of the environment is shown in Fig. 2d.

In order to find a reason for the higher sensitivity of nanoframes compared to nanocages, the mechanism of plasmonic hybridization governing them has been investigated. It is well known that the refractive index sensitivity of nanoparticles depends on the position of the plasmon resonance band^26^. The lower the plasmon resonance band energy, the higher the sensitivity of the nanoparticles. The important point is what factors determine the position of the nanoparticle resonance band?

**Figure 3:**
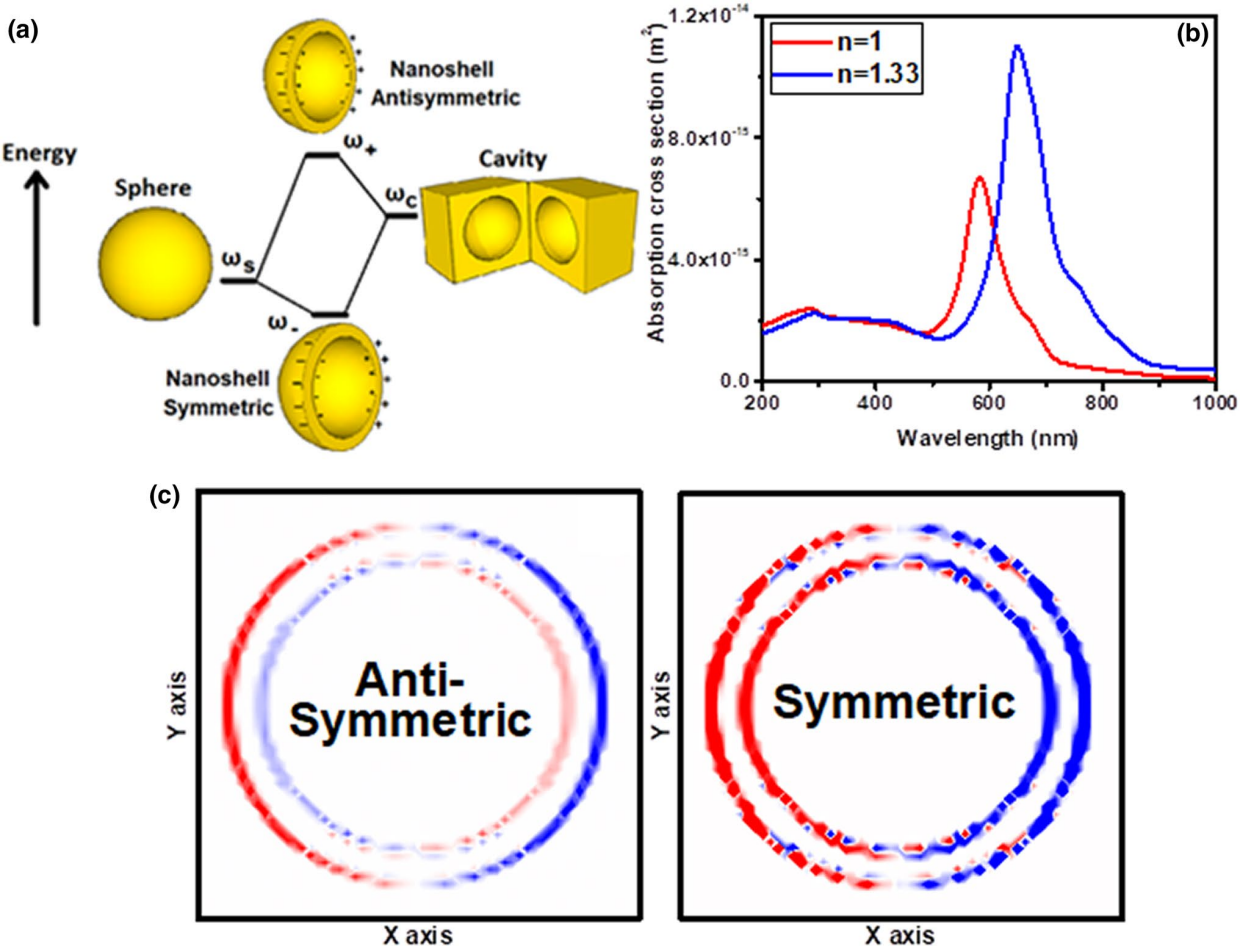
(**a**) Energy-level diagram describing the plasmon hybridization mechanism of nanoshells: Effect of shell thickness on the interaction between a sphere (resonance frequency, ω_s_) and a cavity plasmon (resonance frequency, ω_c_). There are two modes of plasmonic oscillations: antisymmetric mode with the resonance frequency ω_+_ and symmetric mode with the resonance frequency ω_−_. (**b**) Absorption cross section of versus refractive index of surrounding medium, n. (**c**) Charge density distribution of the spherical nanoshell at its symmetric and asymmetric resonance modes. Browser-based version of SketchUp, https://app.sketchup.com, and Origin 2020 version, https://www.originlab.com, have been used for drawing images and making figures, respectively.

According to the model proposed by Prodan et al. for the case of spherical nanoshells, the simplest structure in which the plasmon hybridization mechanism prevails, two resonance modes of symmetric and asymmetric are produced at low and high frequencies in interaction with light, respectively^18^ (Fig. 3a). Here, the absorption spectra of a spherical nanoshell with a shell thickness of 4.5 nm and an overall size of 50 nm are calculated (Fig. 3b). As the plasmon hybridization model predicts, two resonance modes occur at the wavelengths of 283 and 583 nm (Fig. 3b). Investigation of the nanoparticle charge density distribution at resonance modes shows symmetric and asymmetric oscillations at 583 and 283 nm wavelengths, respectively (Fig. 3c). Increasing the refractive index of the environment, which nanoparticle is embedded, leads to red-shift of its absorption spectrum, where the symmetric plasmonic peak experiences greater displacement than the asymmetric peak (Fig. 3b).

**Figure 4:**
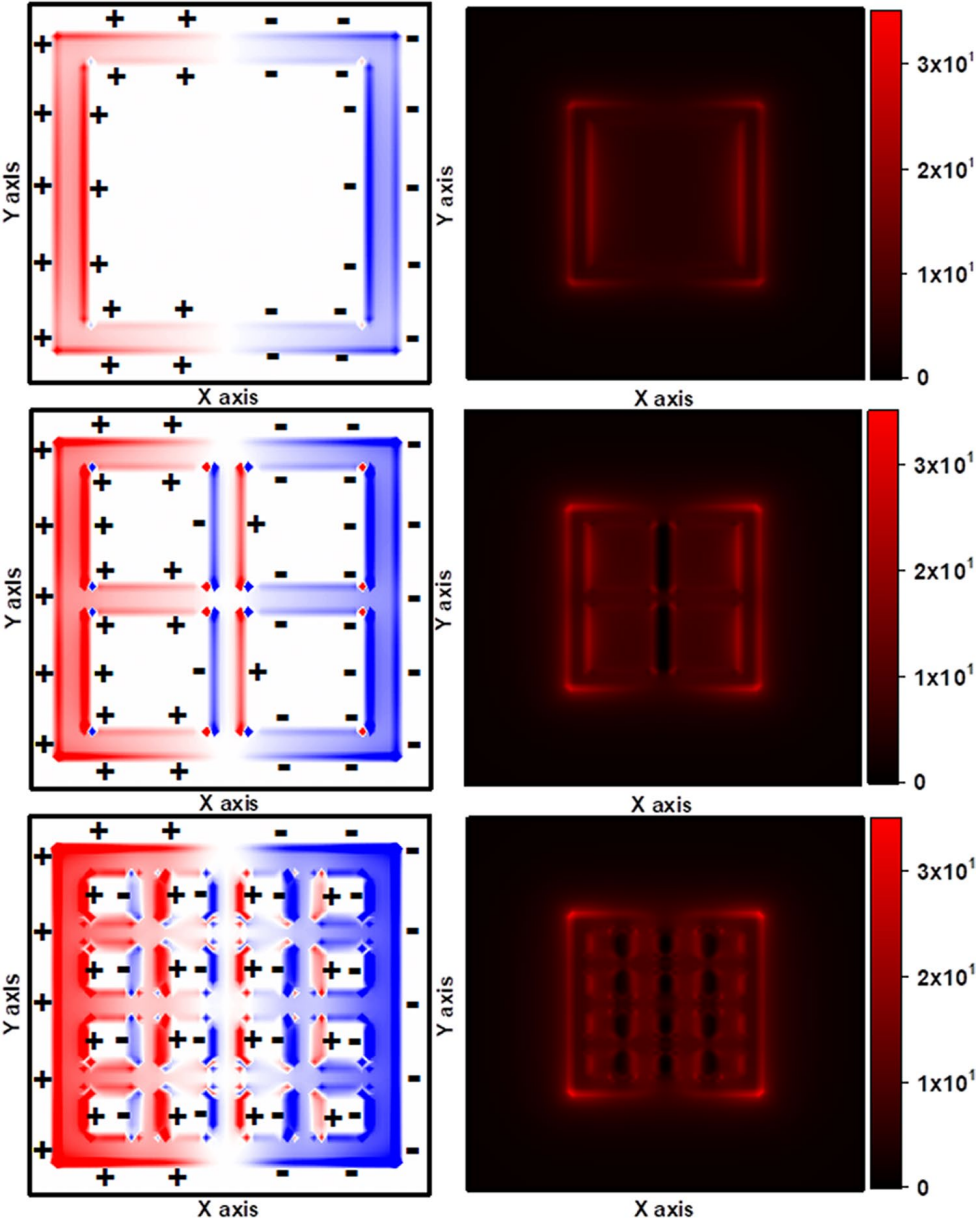
Charge density and near-field distribution of the cubic nanocage with q = 2 and 4 and nanoframe at the resonance wavelength. Symmetric and asymmetric modes of plasmon hybridization of facing surfaces are evident from the charge density map. Browser-based version of SketchUp, https://app.sketchup.com, and Origin 2020 version, https://www.originlab.com, have been used for drawing images and making figures, respectively.

Figure 4 shows the charge density and near-field distribution of nanocages compared to nanoframes in the dipolar resonant mode. A significant phenomenon that can be observed in the charge density distribution of nanocages, unlike nanoframes, is the simultaneous existence of asymmetric and symmetric oscillations in the resonant mode. Asymmetric oscillation, which is considered a dark mode, not only does not couple to the far-field radiation, but also shows less sensitivity to changes in the refractive index of the environment compared to light modes. The near-field distribution profile clearly shows the lack of generation of strong plasmonic fields around the walls covered with dark modes (asymmetric oscillations). Here, the outcome of the behavior of both oscillations determines the position of the nanoparticle plasmon resonance band and its sensitivity. As the refractive index of the environment increases, asymmetric oscillations, which have a low tendency to wavelength displacement, act as a limiting factor for nanoparticle sensitivity. The nanocages with 4*4 cavities array produce more asymmetric oscillations compared to the 2*2 cavities array in the resonant mode. The presence of these asymmetric oscillations in the resonance mode can be a factor for the lower sensitivity of nanocages than nanoframes.

**Figure 5:**
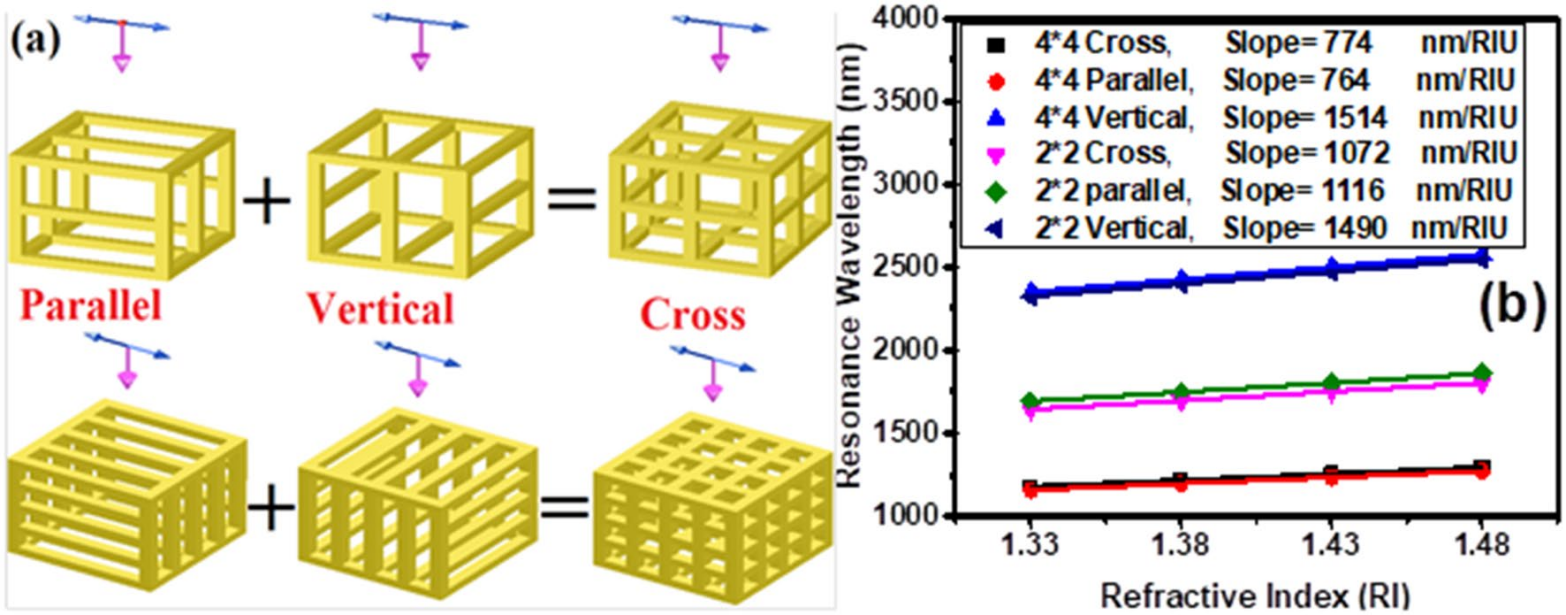
(**a**) Schematic views of divided nanocages into two structures of vertical and parallel, which have walls perpendicular and parallel to the direction of incident light polarization, respectively. (**b**) Resonance wavelength shift of 2*2 and 4*4 nanocages and their divided structures, vertical and parallel, versus different refractive index of surrounding medium. Browser-based version of SketchUp, https://app.sketchup.com, and Origin 2020 version, https://www.originlab.com, have been used for drawing images and making figures, respectively.

On the other hand, a more in-depth study of the parameters affecting the plasmon resonance band of hybrid nanoparticles showed that the restoring force acts as a determining parameter^27^. As mentioned earlier, the refractive index sensitivity of the nanoparticle is directly related to its plasmonic peak position. Therefore, the effect of restoring force on the sensitivity of nanocages is investigated in Fig. 5. For this purpose, nanocages are divided into two structures, vertical and parallel, which have walls perpendicular to and parallel with respect to the polarization of incident light, respectively (see Fig. 5a). Investigation of their sensitivity shows that plasmonic peaks of parallel structures occur at shorter wavelengths and are less sensitive compared to vertical structures (Fig. 5b). In fact, in a parallel structure, the presence of walls parallel to the polarization direction of light contributes to the easier displacement of electrons during dipolar resonance and, consequently, to remain the plasmon energies at higher levels. In contrast, in vertical structures the strength of surface charges is reduced by the walls perpendicular to the direction of incident light polarization, leading to a reduction in the restoring force and thereby lowering the energy of plasmons. Similarly, as the number of these walls perpendicular to the polarity is increased, the nanoparticle plasmon peak is more likely to occur at higher wavelengths. This phenomenon is so prevalent in the nanoparticles that the vertical structures of the nanocages showed greater sensitivities than nanoframes (Albeit minor). In the following, nanoframes have been further investigated due to their high sensitivity and low dependence on light polarization compared to nanocages.

**Figure 6:**
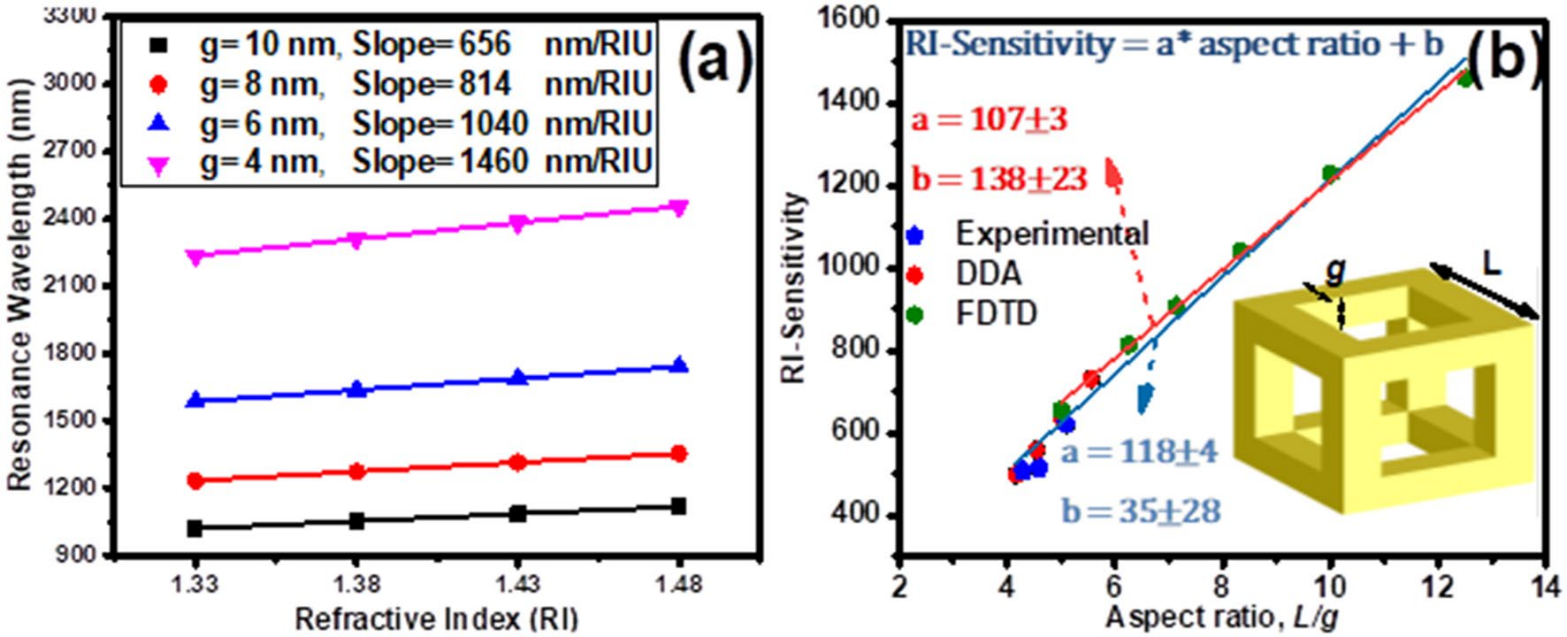
(**a**) Resonance wavelength shift of cubic nanoframes with various wall thickness (g) versus different refractive index of surrounding medium. (**b**) The RI-Sensitivity shift of cubic nanoframes versus different aspect ratio, R (wall length (L) -to- wall thickness (g) ratio). Fitted with the data of the linear equation S = a × R + b. Browser-based version of SketchUp, https://app.sketchup.com, and Origin 2020 version, https://www.originlab.com, have been used for drawing images and making figures, respectively.

According to the mentioned results, further optimization has been performed on the wall thickness of nanoframes with a wall length of 50 nm in order to achieve a RI-sensitivity estimation equation over a wide range of aspect ratio, R (ratio of wall length (*L*) to wall thickness (*g*)). Also, to evaluate the overlapping of simulation results with experimental results, a comparative study has been presented. Figure 6a shows RI- sensitivity analysis of nanoframes with the wall thicknesses of 4, 6, 8, and 10 nm. The wall thickness reduction increases the RI-sensitivity of nanoframes from 656 to 1460 nm/RIU due to the strengthening of facing surfaces plasmons. RI-sensitivity of the nanoframes is reasonably correlated and linearly increased with the aspect ratio. Fitting the RI-sensitivity versus the aspect ratio, gives the equation of $S = \frac{\delta \lambda }{{\delta n}} = \left( {107 \pm 3} \right)\frac{L}{g} + \left( {138 \pm 23} \right)$, for nanoframes with aspect ratio in the range of 5–12.5 (Fig. 6b (red fit line)). Mahmoud et al., in an experimental and DDA theory has studied on the nanoframes^24^, have presented the equation of $S = \frac{\delta \lambda }{{\delta n}} = 164\frac{L}{g} - 180$, which shows the RI-sensitivity how changes for different aspect ratios in the range of 4–5.5. Our simulation results in the common aspect ratio range of 5–5.5 are in good agreement with the results of their work. At higher values of aspect ratio, 5.5 to 12.5, the equation proposed by Mahmoud et al. have not been accurately predicted and are not in agreement with the results obtained in this paper. In fact, the equation previously presented in this paper also does not provide correct estimates for aspect ratio values below 5, and the more aspect ratio reduces,the more the predicted error increases. Hence, using the results of experimental and DDA theory and the FDTD simulation results obtained in this paper, a more general equation for aspect ratio in the wide range of 4–12.5 is proposed which can properly predicts the nanoframes RI-sensitivity (Fig. 6b (blue fit line)):

2 $$S = \frac{\delta \lambda }{{\delta n}} = \left( {118 \pm 4} \right)\frac{L}{g} + \left( {35 \pm 28} \right)$$

## Conclusion

In summary, we have shown the effect of plasmon hybridization mechanism on the LSPR properties and RI-sensitivity of several single metallic nanostructures: SiO_2_@Au core–shell nanocube, Au nanocage, and nanoframe characterized by FDTD simulation. We have used charge density distribution calculations to show that the simultaneous presence of symmetric and asymmetric oscillations in nanocages in the plasmon resonance mode can be a limiting factor in their RI-sensitivity. We have also shown that the restoring force in dipolar resonance acts as a parameter determining the sensitivity of nanocages, since the sensitivity of nanoparticles is directly related to the position of their plasmon resonance band. By studying the effect of the array of cavities in nanocages, we have shown that with the reduction of the asymmetric oscillations occuring in the resonant mode of the nanocage, the sensitivity of the refractive index of its surroundings becomes more prominent. Also, the presence of walls parallel with respect to the polarization of light contributes to the easier displacement of electrons during dipolar resonance and, consequently, maintains the plasmon energies at high level. These results could somehow advance the understanding of why nanocages are less sensitive compared to nanoframes. Nanocages with 1*1 cavity array, which has the same structure as the nanoframe, shows the most sensitivity due to the generation pure symmetrical oscillations in the resonant mode and having a reduced restoring force. In this way, by optimizing and examining the wall thickness of the nanoframe structure, in overlap with the previously reported experimental results, we have presented a linear equation for estimating the sensitivity as a function of aspect ratio, R. This equation predicts the sensitivity of 1460 nm/RIU for nanoframes with the aspect ratio of 12.5, which is more than fivefold of their solid counterparts. The results of this paper provide a useful recipe for fabricating more sensitive nanosensors for medical diagnostics.

## Methods

We evaluated the potential use of single gold nanoparticles as sensors by employing the FDTD computational method using OptiFDTD commercial software. FDTD permits a solution of the Maxwell’s curl equations via subbing all time and space derivatives by finite time and space differences^28^. This method enables us to obtain the distribution of electromagnetic fields approximate to metallic objects and hence gives an algorithm for calculating absorption and scattering cross-sections of different objects. Since the solution depends on boundary conditions, the field distribution near the object is determined by their shape, their martial and their environment. Therefore, FDTD is vastly used to study the near field electromagnetic response of metallic nanoparticles. To calculate RI sensitivity of a metallic nanoparticle we assume that it is illuminated by a uniform downward-directed linearly polarized electromagnetic wave, propagating in the z-direction. The polarization axis is assumed to be along the x-direction. The wave scattered from the surface of the nanoparticle. In order to avoid the backscattering effects from boundaries we employed Perfectly Matched Layer (PML) boundary conditions^29^. By this we mean that the impedance matching conditions is completely satisfied in all directions through the boundaries. The starting point of calculation of RI sensitivity of metallic nanoparticles lies on the concept of the absorption cross-section. The absorption cross-section is obtained by^30^:

3 $$\sigma_{abs} = \frac{{P_{abs} }}{{I_{inc} }},$$

where I_inc_ is the incident (landing) wave intensity and power absorbed, P_abs_ is defined by^30^:

4 $$P_{abs} = \frac{1}{2}\Re \left( {\int_{r} {E_{abs} \times H^{ * }_{abs} .\hat{n}da} } \right),$$

here $E_{abs}$ and $H_{abs}$ are absorption electric and magnetic fields, respectively. The sensitivity of the nanoparticles to the variation of RI of their surrounding medium is defined by the ratio of the resonance wavelength shift of absorption cross-section ($\delta \lambda$) due to the variation of embedding medium RI ($\delta n$):

5 $$S = \frac{\delta \lambda }{{\delta }}$$

In spite of the fact that the plasmonic performance of silver nanoparticles is better than the gold nanoparticles, their application to bio-sensors is restricted by their low chemical stability and also their bio-incompatibility^23^. Hence the gold nanoparticles are superior to silver nanoparticles and in the following, we concentrate on the sensitivity of gold nanoparticles fabricated by Johnson and Christy’s method^31^.

## Supplementary Information

Supplementary Information.
