# Supplementary material for: Ultrahigh sensitive refractive index nanosensors based on nanoshells, nanocages and nanoframes: effects of plasmon hybridization and restoring force
Source: Sci Rep. 2021 Jan 22;11:2065. doi: 10.1038/s41598-021-81578-w (PMC7822811; doi:10.1038/s41598-021-81578-w)
Supplement: Supplementary file 1 — Supplementary Information. [file 41598_2021_81578_MOESM1_ESM.docx]

**Ultrahigh sensitive refractive index nanosensors based on nanoshells, nanocages and nanoframes: Effects of plasmon hybridization and restoring force**

MirKazem Omrani ^*1^, Hamidreza Mohammadi ^*1, 2^, Hamidreza Fallah ^1, 2^

1. Department of Physics, University of Isfahan, P.O. Box 81746-7344, Isfahan, Iran
2. Quantum Optics Research Group, University of Isfahan, Isfahan, Iran

- Corresponding authors email: [m.k.omrani@sci.ui.ac.ir](mailto:m.k.omrani@sci.ui.ac.ir) (M. K. Omrani) [hr.mohammadi@sci.ui.ac.ir](mailto:hr.mohammadi@sci.ui.ac.ir) (H. Mohammadi)

**Fig. S1.**Charge density distribution of the cubic nanosolid and nanoshell with T=4 at their resonance wavelength. Origin 2020 version, <https://www.originlab.com> , has been used for making figures.

**Fig. S2.** (a) Near field profiles of cubic nanosolid and nanoshell (silica core-gold shell) at peak plasmon resonance. (b) Near field enhancement as a function of distance from the nanoparticle surface at the plasmon resonance wavelength in X axis line (Y location assumed at 0 and 25 nm). (c) Localized surface plasmon resonance (LSPR) quenching; Plasmon field radiation versus time. Origin 2020 version, <https://www.originlab.com> , has been used for making figures.
